# Mix-Proportion Evaluation and Microstructural Characteristics of NaOH-Na_2_SO_4_ Composite-Activated Fly Ash-Based Grouting Materials

**DOI:** 10.3390/ma19183854

**Published:** 2026-09-10

**Authors:** Mengxin Xu, Feng Ju, Meng Xiao, Tengfei Wang, Dong Wang, Lidong Yin, Yingbo Wang, Lu Si, Dongming Yang

**Affiliations:** 1 School of Mechanics and Civil Engineering, China University of Mining and Technology, Xuzhou 221116, China; ts23030019a31@cumt.edu.cn (M.X.); 15255791521@163.com (Y.W.); ts23030012a31@cumt.edu.cn (L.S.); dongmingyang11@outlook.com (D.Y.); 2 State Key Laboratory of Intelligent Construction and Healthy Operation and Maintenance of Deep Underground Engineering, China University of Mining and Technology, Xuzhou 221116, China; m.xiao@cumt.edu.cn (M.X.); wangtf0623@cumt.edu.cn (T.W.); mechanics@cumt.edu.cn (D.W.); yinlidong532@163.com (L.Y.)

**Keywords:** fly ash, grouting material, NaOH-Na_2_SO_4_ composite activation, mix-proportion evaluation, microstructure

## Abstract

**Highlights:**

- Water-to-solid ratio, total activator dosage, and NaOH:Na_2_SO_4_ affected the fresh and hardened properties.
- The preferred mixture had a water-to-solid ratio of 0.55, a total activator dosage of 5%, and NaOH:Na_2_SO_4_ = 2:1.
- NaOH:Na_2_SO_4_ = 2:1 was associated with a denser 7 d microstructure and fewer pores and cracks.
- Provides a basis for mix-proportion selection of fly ash-based grouting materials.

**Abstract:**

To improve fly ash utilization in mine grouting materials and address the slow reaction and limited early-age strength of fly ash-based binders, a fly ash-based grout was prepared using fly ash and S95-grade ground granulated blast-furnace slag at a mass ratio of 70:30 and activated with NaOH-Na_2_SO_4_. A full-factorial experiment evaluated the effects of water-to-solid ratio, total activator dosage, and NaOH:Na_2_SO_4_ on fluidity, bleeding rate, stone formation rate, and compressive strength at 7 and 28 d. Representative 7 d specimens were characterized by SEM, EDS, and XRD. Increasing the water-to-solid ratio improved fluidity but increased the bleeding rate and reduced the stone formation rate and compressive strength. The preferred mixture had a water-to-solid ratio of 0.55, a total activator dosage of 5%, and NaOH:Na_2_SO_4_ = 2:1. It exhibited a fluidity of 195.0 mm, a bleeding rate of 1.70%, a stone formation rate of 99.50%, and compressive strengths of 8.5 MPa at 7 d and 11.3 MPa at 28 d. At NaOH:Na_2_SO_4_ = 2:1, the specimen showed a denser microstructure with fewer pores and cracks, a local Ca/Si ratio of approximately 0.94, and more evident reaction-product features. The results provide an experimental basis for mix-proportion selection of fly ash-based grouting materials.

## 1. Introduction

Grouting reinforcement is widely used in coal mines for goaf filling, reinforcement of fractured surrounding rock, fracture sealing, and mine water control. Its engineering performance is closely related to grout fluidity, bleeding stability, setting and hardening behavior, and the strength of the hardened grout. Common grouting materials can be broadly classified as cement-based, chemical or polymer-based, and solid-waste-based systems. Cement-based grouts are widely used because of their mature preparation procedures and relatively high hardened strength. However, their high cement consumption increases material cost and embodied energy, while bleeding and particle sedimentation may occur at high water-to-cement ratios. Chemical and polymer grouts generally offer adjustable viscosity and setting time and good injectability into fine fractures, but their relatively high cost and potential durability and environmental concerns restrict large-scale application [1,2]. Industrial solid wastes, including fly ash, ground granulated blast-furnace slag (GGBFS), and coal gangue, have therefore been introduced as partial substitutes for cement in mine grouting and underground backfilling. Aluminosilicate solid wastes are also increasingly used in geopolymer and alkali-activated grouts to reduce cement consumption and improve solid-waste utilization [3]. Zhang et al. [4] proposed a gangue grouting backfilling method for post-mining voids, and Zhou et al. [5] investigated the groutability of low-calcium fly ash-cement slurry. Nevertheless, the low ambient-temperature reactivity of fly ash often makes it difficult to balance grout fluidity, bleeding stability, and early-age strength.

Previous studies have attempted to improve grout performance by adjusting binder composition and water-to-solid ratio, incorporating accelerators or early-strength agents, using two-component systems, and introducing nanomaterials. Tong et al. [6] investigated grouting and backfilling materials containing a high proportion of fly ash. Chen and Wang [7] conducted experimental and engineering studies on fly ash grouting materials, while Wu et al. [8] determined the mix proportion of a fly ash-cement two-component grout through orthogonal testing and numerical analysis. More recent studies have focused on cement-free or low-cement grouts mainly composed of industrial solid wastes. Zhang et al. [9] and Xu et al. [10] applied multi-index and multi-objective optimization approaches, respectively, to alkali-activated grouting materials. Guo et al. [11] prepared geopolymer grouts from coal gangue, slag, and fly ash, whereas Li et al. [12] evaluated the fresh and hardened properties of a double-liquid alkali-activated grout. Han et al. [13] modified a cement-based grout using carbon-nanotube-coated fly ash, increasing fluidity and 7 d strength by 21.6% and 7.9%, respectively, while reducing porosity by 11.9%. However, nanomaterials and polymers remain relatively expensive, and some modified systems still rely on cement as the principal binder. In addition, many existing studies have focused on individual properties such as fluidity, setting time, or compressive strength, whereas the combined evaluation of grout fluidity, bleeding resistance, stone formation rate, and strength development has received less attention.

Increasing the proportion of fly ash and GGBFS in grouting materials can reduce cement consumption, improve industrial solid-waste utilization, and lower the energy demand and environmental burden associated with cement production. Global fly ash production exceeds 1 billion tonnes annually [14]. In 2022, China produced approximately 831 million tonnes of fly ash [15], and the cumulative stockpile approached 4 billion tonnes [16,17]. Although fly ash has been used in cement, concrete, and grouting materials [18,19], its aluminosilicate glass phase is relatively stable and reacts slowly at ambient temperature. Chemical activation can promote the dissolution of reactive species from fly ash [20]. Ghafoor et al. [21] and Atis et al. [22] investigated the influence of alkaline activators on the strength of fly ash-based materials. Liu et al. [23] examined the effect of NaOH on the strength of fly ash-cement composites, while Yin et al. [24] quantified the dissolution of Si and Al from low-calcium fly ash in NaOH solution. Regarding Na_2_SO_4_ activation, Lv et al. [25] and Qiu and Wang [26] reported that Na_2_SO_4_ promoted reaction-product formation and improved the hardened structure of fly ash-based materials. Dai et al. [27] compared the fresh properties, reaction process, mechanical properties, and microstructure of Na_2_SO_4_-activated and NaOH-Na_2_SO_4_-activated GGBFS systems, showing that NaOH addition altered the early reaction and hardening behavior of the Na_2_SO_4_-activated system. Yue et al. [28] examined phase evolution in Na_2_SO_4_-activated GGBFS and identified ettringite (AFt) and calcium aluminosilicate hydrates among the principal reaction products. Guo et al. [29] further showed that activator composition affected precursor reaction, reaction-product formation, and compressive strength in coal gangue-slag-fly ash geopolymer grouts. Fernández-Jiménez et al. [30] described the microstructural development of alkali-activated fly ash binders. These studies show that NaOH and Na_2_SO_4_ affect the activation of aluminosilicate precursors through different roles. NaOH provides an alkaline environment that promotes the dissolution of reactive Si and Al species, while Na_2_SO_4_ can promote the formation of sulfate-containing reaction products in Ca-bearing systems. These findings provide the rationale for using NaOH-Na_2_SO_4_ as the composite activator for the fly ash-GGBFS binder investigated in this study.

Most previous studies, however, have focused on fly ash-cement materials, geopolymer concrete, or mortar, and their findings cannot be directly transferred to fly ash-based grouting materials activated with NaOH-Na_2_SO_4_. In these systems, fresh-state performance and hardened strength are jointly affected by the water-to-solid ratio, total activator dosage, and NaOH:Na_2_SO_4_. A high water-to-solid ratio may improve fluidity but increase bleeding and reduce the stone formation rate and compressive strength, whereas a low ratio may impair pipeline transport and fracture penetration. The total activator dosage affects the reaction extent of fly ash and GGBFS, while NaOH:Na_2_SO_4_ influences system alkalinity and the formation of sulfate-containing reaction products such as AFt. However, systematic evaluation of the water-to-solid ratio, total activator dosage, and NaOH:Na_2_SO_4_ within the same fly ash-GGBFS grouting system remains limited, particularly when fluidity, bleeding rate, stone formation rate, and compressive strength are considered together. In addition, relatively few studies have examined the microstructural characteristics of this grouting system at different NaOH:Na_2_SO_4_ ratios in relation to its compressive strength.

This study developed a fly ash-based grouting material using fly ash and S95-grade GGBFS at a mass ratio of 70:30, activated with a NaOH-Na_2_SO_4_ system. A full-factorial experiment was conducted to evaluate the effects of water-to-solid ratio, total activator dosage, and NaOH:Na_2_SO_4_ on fluidity, bleeding rate, stone formation rate, and compressive strength at 7 and 28 d. The preferred mixture was selected by jointly considering fresh-state performance and hardened strength. Representative specimens with different NaOH:Na_2_SO_4_ ratios were subsequently characterized by scanning electron microscopy, energy-dispersive spectroscopy, and X-ray diffraction to examine their microstructure, local elemental composition, and mineral phases, and to relate the observed microstructural differences to compressive strength.

## 2. Materials and Methods

### 2.1. Raw Materials

The raw materials used in this study were fly ash, S95-grade GGBFS, NaOH, Na_2_SO_4_, and water. The fly ash was obtained from Shang’an Power Plant in Shijiazhuang, Hebei Province, China, and was a gray fine powder without visible agglomeration. The GGBFS was obtained from Hebei Jingye Steel Co., Ltd., Shijiazhuang, China. NaOH and Na_2_SO_4_ were supplied by Xilong Scientific Co., Ltd., Shantou, China. NaOH was supplied as white granules with a purity of at least 96%, whereas Na_2_SO_4_ was an analytical-grade powder with a purity of at least 99%. Laboratory tap water was used throughout the experiments. The basic properties of the fly ash, including particle size distribution, mineral composition, and chemical composition, were characterized using laser particle size analysis, X-ray diffraction (XRD), and X-ray fluorescence (XRF), respectively.

### 2.2. Mix Proportion Design

Fly ash used as the sole binder generally exhibits slow reaction and limited early-age strength under ambient curing conditions [31,32]. Therefore, a binder composed of fly ash and GGBFS was adopted to improve early reaction and strength development [33,34]. Based on preliminary tests, the binder composition was fixed at 70 wt% fly ash and 30 wt% GGBFS, and NaOH and Na_2_SO_4_ were used as the composite activator.

Based on preliminary tests and previous studies on chemically activated fly ash-based materials, a full-factorial experiment was conducted to evaluate the effects of water-to-solid ratio, total activator dosage, and NaOH:Na_2_SO_4_ on the properties of the fly ash-based grouting materials [35]. The investigated ranges were selected to provide different levels of the three mix-proportion parameters for systematic comparison within the present binder system. The water-to-solid ratios were 0.50, 0.55, and 0.60. The total activator dosages were 4%, 5%, and 6% of the total binder mass. The total activator dosage was defined as the combined mass of NaOH and Na_2_SO_4_ divided by the total mass of fly ash and GGBFS. NaOH:Na_2_SO_4_ was set at 3:1, 2:1, 1:1, 1:2, and 1:3. All NaOH:Na_2_SO_4_ values reported in this study are mass ratios. The complete combination of these levels resulted in 45 mixtures, as listed in Table 1.

### 2.3. Specimen Preparation and Curing

**Figure 1:**
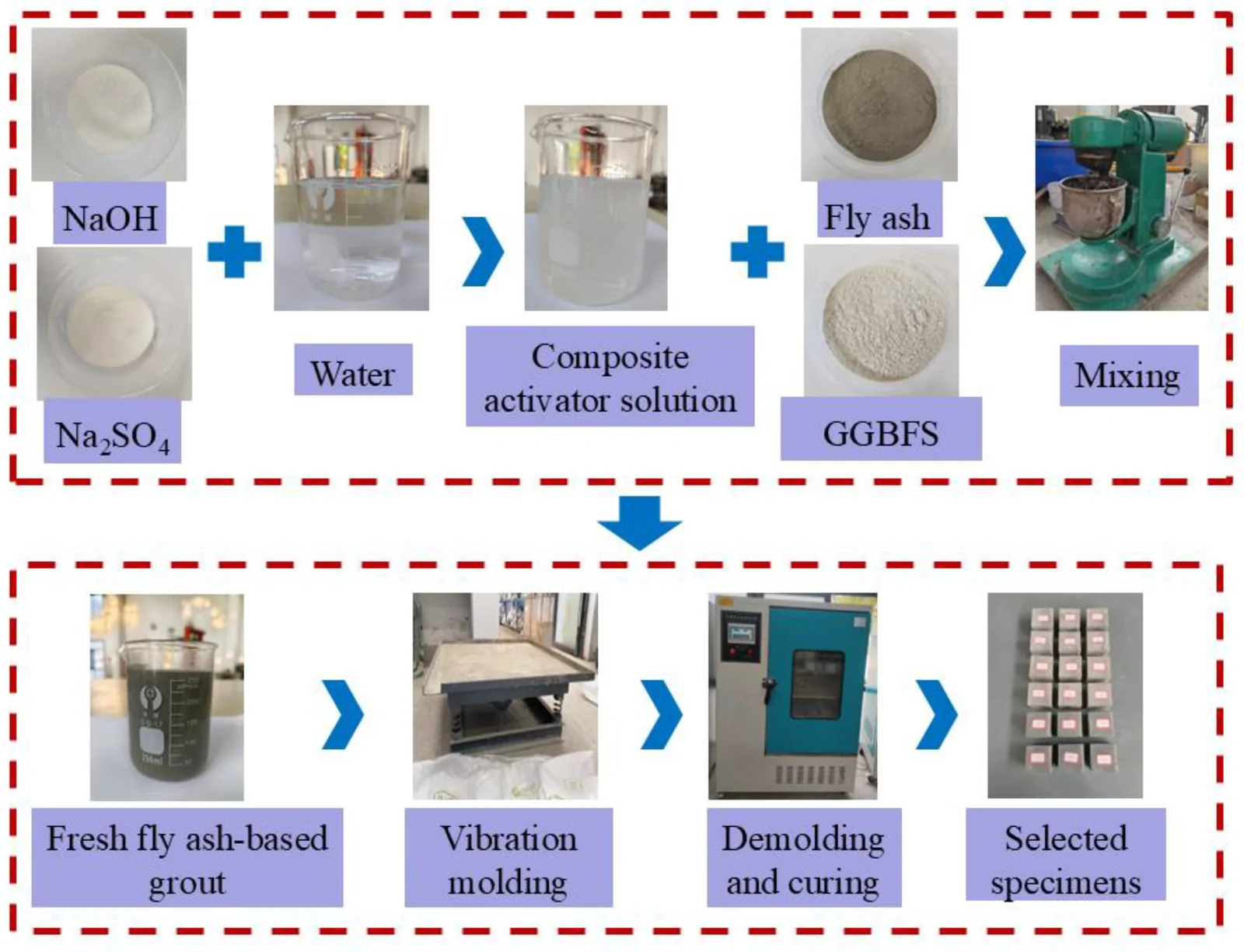
Specimen preparation process for the fly ash-based grouting materials.

The preparation process of the fly ash-based grouting material specimens is shown in Figure 1. It mainly included raw material weighing, mixing, vibration molding, demolding, and curing [36]. The specific preparation procedure was as follows:

First, raw material weighing. According to the mix proportions listed in Table 1, fly ash, GGBFS, NaOH, Na_2_SO_4_, and water were weighed separately.

Second, mixing. NaOH and Na_2_SO_4_ were first dissolved in water and stirred for 3 min to prepare a homogeneous activator solution. Fly ash and GGBFS were then dry-mixed until uniform, after which the activator solution was added. The mixture was stirred for a further 5 min to obtain a homogeneous grout.

Third, vibration molding. The fresh grout was cast into cubic molds measuring 70.7 mm × 70.7 mm × 70.7 mm and compacted on a vibrating table until no visible bubbles emerged from the surface.

After 24 h, the specimens were demolded and transferred to a curing chamber maintained at 20 ± 2 °C and 98% relative humidity. Specimens for compressive strength testing were cured for 7 and 28 d. Specimens for microstructural characterization were prepared using the same procedure and sampled at 7 d. All tests were conducted with three parallel measurements or specimens, and the reported results represent the average values of repeated tests.

### 2.4. Test Methods

#### 2.4.1. Workability Tests

Fluidity, bleeding rate, and stone formation rate were measured to evaluate the flowability, bleeding stability, and volume retention of the fly ash-based grouts [37]. Fluidity was evaluated using the spread diameter of the fresh grout. The grout was poured into the test mold and allowed to spread freely after the mold was lifted. Two perpendicular spread diameters were measured, and their average was reported as the fluidity.

The bleeding rate was used to evaluate the stability of the grout after standing. Immediately after the mixing procedure described in Section 2.3, the fresh grout was transferred into a 250 mL graduated cylinder. The initial grout volume, *V*_0_, was recorded, and the grout was allowed to stand for 2 h. The volume of separated water, *V_i_*, was then measured. Each test was repeated three times, and the mean value was used.

(1) $$R_{s}=\frac{V_{i}}{V_{0}}\times 100\%$$

where *R_s_* is the bleeding rate (%), *V_i_* is the volume of separated water after 2 h (mL), and *V*_0_ is the initial grout volume (mL).

The stone formation rate was used to evaluate the volume retained after grout hardening. The stone formation rate, *β*, was defined as the ratio of the hardened grout volume, *V*_2_, to the initial grout volume, *V*_1_. After hardening, the grout height was measured at several positions and averaged. The stone formation rate was calculated using Equation (2):

(2) $$\beta =\frac{V_{2}}{V_{1}}\times 100\%$$

where *β* is the stone formation rate (%), *V*_2_ is the hardened grout volume (cm^3^), and *V*_1_ is the initial grout volume (cm^3^).

Considering the practical requirements of grouting materials for pumpability, slurry stability, and hardened-grout integrity, and referring to previous studies on fly ash-based grouting materials [38], this study adopted a fluidity of ≥180 mm, a bleeding rate of <5%, and a stone formation rate of ≥90% as the criteria for mix-proportion screening. The fluidity criterion was used to ensure sufficient flowability for grout transportation and penetration, while the bleeding rate and stone formation rate were adopted to evaluate slurry stability and hardened-grout integrity. These criteria were used for comparative screening within the investigated experimental range.

#### 2.4.2. Compressive Strength Test

Compressive strength was used to evaluate the mechanical performance of the hardened grout. Specimens were prepared and cured as described in Section 2.3. Compressive strength was measured using 70.7 mm × 70.7 mm × 70.7 mm cubic specimens cured for 7 and 28 d. The specimens were tested at a displacement rate of 0.3 mm/min. Three specimens were tested for each mixture and curing age, and the mean value was reported.

#### 2.4.3. Microstructural Characterization

Representative specimens were selected for scanning electron microscopy (SEM), energy-dispersive spectroscopy (EDS), and X-ray diffraction (XRD) analyses to examine their morphology, local elemental composition, and mineral phases. All specimens used for microstructural characterization were cured for 7 d. Internal fragments were collected, immersed in anhydrous ethanol to terminate further reaction, and dried before testing. SEM-EDS analysis was performed using a ZEISS-Sigma 360 field-emission scanning electron microscope (Zeiss, Oberkochen, Germany) equipped with an energy-dispersive spectroscopy detector. SEM was used to examine pores, cracks, and reaction-product morphology, whereas EDS was used to determine the local contents of Si, Al, O, and Ca in selected regions. XRD analysis was performed using a Bruker D8 Advance diffractometer (Bruker, Karlsruhe, Germany). XRD patterns were collected over a 2θ range of 5–90° at a scanning rate of 2°/min.

## 3. Results and Discussion

### 3.1. Characterization of Raw Materials

**Figure 2:**
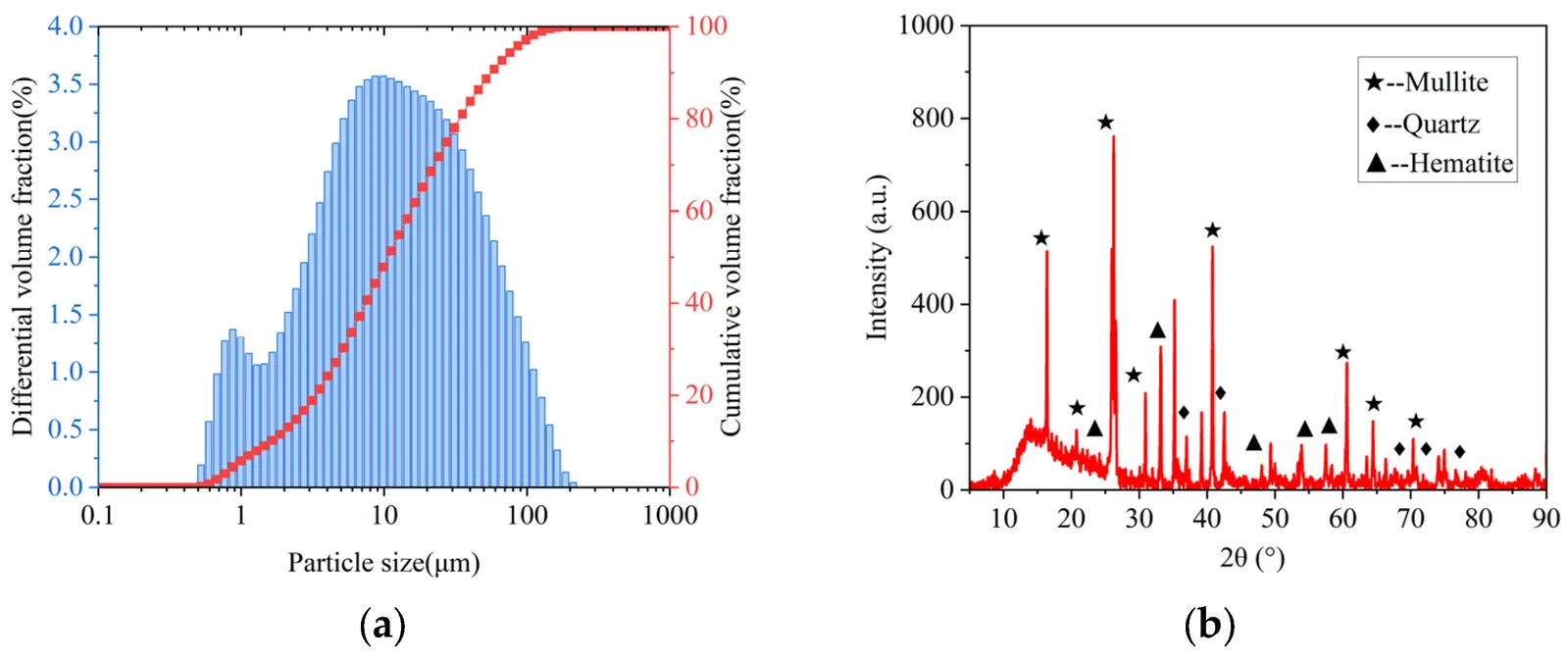
Particle size distribution and XRD pattern of fly ash: (**a**) particle size distribution; (**b**) XRD pattern.

The particle size distribution and mineral composition of the fly ash are presented in Figure 2. The particle size distribution of the fly ash is shown in Figure 2a. Particles smaller than 100 μm accounted for 97.18%, indicating that the fly ash had a predominantly fine particle-size distribution. This distribution may favor particle packing in the grout [39]. The XRD pattern of the fly ash is shown in Figure 2b. As shown in the figure, the fly ash was mainly composed of mullite, quartz, and hematite. Among them, mullite is a stable crystalline phase formed during high-temperature treatment and may remain as a relatively inert crystalline component in the hardened grout. Quartz has relatively low chemical reactivity; however, fine quartz particles can be dispersed within the system and provide a certain pore-filling effect.

The chemical composition of the fly ash determined by XRF is listed in Table 2. As shown in the table, the fly ash contained high contents of SiO_2_ and Al_2_O_3_, with mass fractions of 50.397% and 38.823%, respectively. The contents of Fe_2_O_3_ and CaO were 3.713% and 1.832%, respectively, while the other components were present in relatively low amounts. SiO_2_ and Al_2_O_3_ provide the main silicon and aluminum sources for the subsequent activation reaction of the fly ash-based grouting material [40]. The silicate and aluminate components in fly ash can dissolve under alkaline conditions and participate in the formation of aluminosilicate-related reaction products.

### 3.2. Workability Analysis

The workability of the fly ash-based grouting materials was evaluated using fluidity, bleeding rate, and stone formation rate, and the results are discussed below.

#### 3.2.1. Fluidity Analysis

**Figure 3:**
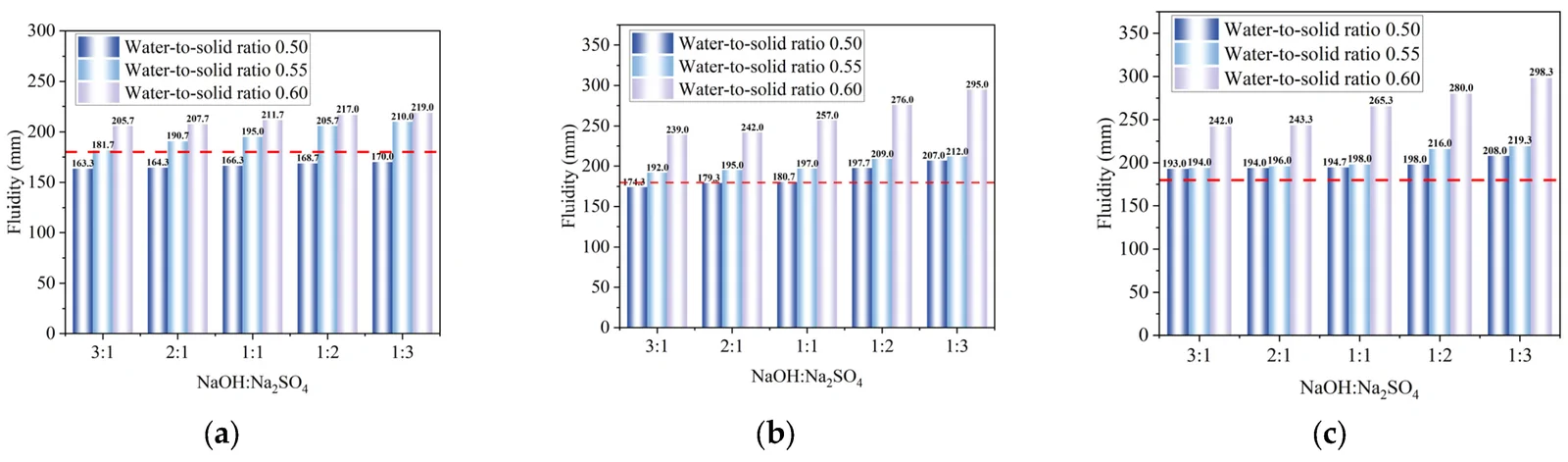
Fluidity variation of the fly ash-based grouting materials: (**a**) total activator dosage of 6%; (**b**) total activator dosage of 5%; (**c**) total activator dosage of 4%.

The fluidity variation of the fly ash-based grouting materials under different mix-proportion conditions is shown in Figure 3. The water-to-solid ratio, total activator dosage, and NaOH:Na_2_SO_4_ all affected grout fluidity. At fixed total activator dosage and NaOH:Na_2_SO_4_ ratio, fluidity increased with the water-to-solid ratio. For example, with a total activator dosage of 6% and a NaOH:Na_2_SO_4_ ratio of 3:1, increasing the water-to-solid ratio from 0.50 to 0.60 increased the fluidity from 163.3 mm to 205.7 mm. The fluidity increased by approximately 26.0%, indicating that water content played an important role in regulating the flow behavior of the grout system. This trend was mainly attributed to the increase in free water, which enlarged the interparticle spacing, enhanced lubrication, weakened particle interactions, and consequently reduced grout viscosity [41,42].

Lower total activator dosage or relative NaOH content generally increased fluidity, possibly because slower early reactions and weaker particle agglomeration delayed viscosity build-up. At a water-to-solid ratio of 0.60 and NaOH:Na_2_SO_4_ = 3:1, reducing the activator dosage from 6% to 4% increased fluidity from 205.7 mm to 242.0 mm. Similarly, at a water-to-solid ratio of 0.60 and a total activator dosage of 5%, changing NaOH:Na_2_SO_4_ from 3:1 to 1:3 increased fluidity from 239.0 mm to 295.0 mm. Based on the 180 mm fluidity criterion, all mixtures with a water-to-solid ratio of 0.50 and 6% activator failed to meet the requirement, as did those with 5% activator and NaOH:Na_2_SO_4_ ratios of 3:1 or 2:1.

#### 3.2.2. Bleeding Rate Analysis

**Figure 4:**
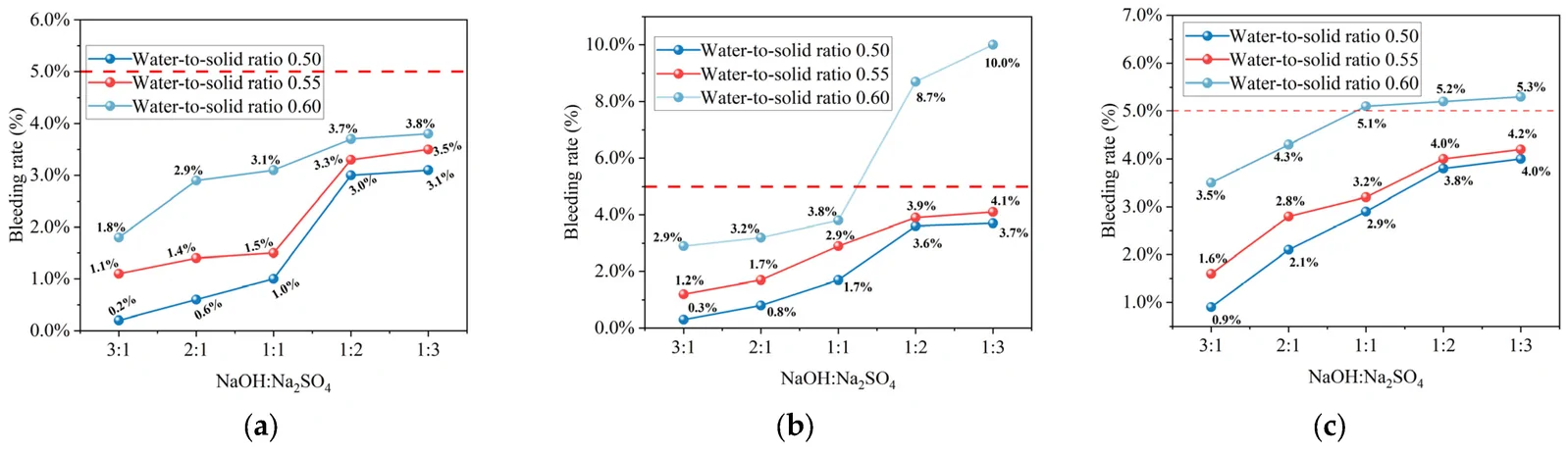
Bleeding rate of the fly ash-based grouting materials: (**a**) total activator dosage of 6%; (**b**) total activator dosage of 5%; (**c**) total activator dosage of 4%.

The bleeding rate variation of the fly ash-based grouting materials under different mix-proportion conditions is shown in Figure 4. The water-to-solid ratio, total activator dosage, and NaOH:Na_2_SO_4_ all affected the grout stability after standing. At fixed total activator dosage and NaOH:Na_2_SO_4_ ratio, the bleeding rate increased with the water-to-solid ratio. For example, at a total activator dosage of 6% and a NaOH:Na_2_SO_4_ ratio of 3:1, increasing the water-to-solid ratio from 0.50 to 0.55 and 0.60 increased the bleeding rate from 0.20% to 1.10% and 1.80%, respectively. Increasing the water-to-solid ratio diluted both the solid phase and the activator solution. Consequently, excess water could not be effectively retained by the developing grout structure and was more likely to separate during standing. This result is consistent with previous studies, which reported that the water-to-solid ratio influences the fresh-state properties and mechanical performance of alkali-activated materials [9,10]. At fixed water-to-solid ratio and NaOH:Na_2_SO_4_ ratio, decreasing the total activator dosage generally increased the bleeding rate. At a water-to-solid ratio of 0.60 and a NaOH:Na_2_SO_4_ ratio of 3:1, the bleeding rate increased from 1.80% to 2.90% and 3.50% as the total activator dosage decreased from 6% to 5% and 4%, respectively. This trend suggests that a lower total activator dosage limited precursor dissolution and early reaction-product formation, thereby reducing the ability of the developing grout structure to retain free water. In the NaOH-Na_2_SO_4_ activated fly ash-based grouting system, sufficient activator dosage is required to promote the dissolution of reactive Si and Al species from the precursors and the subsequent development of binding products. A reduced activator dosage may therefore weaken the early structural development of the grout and decrease its ability to retain water during standing.

The NaOH:Na_2_SO_4_ ratio also affected the bleeding rate. At fixed water-to-solid ratio and total activator dosage, the bleeding rate generally increased as the relative NaOH content decreased. For example, at a water-to-solid ratio of 0.55 and a total activator dosage of 6%, the bleeding rate increased from 1.10% to 3.50% when the NaOH:Na_2_SO_4_ ratio changed from 3:1 to 1:3. At a water-to-solid ratio of 0.60 and a total activator dosage of 5%, the corresponding increase was from 2.90% to 10.00%. This trend suggests that insufficient alkalinity limited precursor dissolution and early reaction-product formation, thereby weakening the ability of the grout structure to retain free water. Similar effects of activator composition on precursor reaction have been reported in alkali-activated GGBFS and multi-solid-waste grouting systems [27,29]. According to the screening requirement of a bleeding rate below 5%, mixtures with a water-to-solid ratio of 0.60 and low relative NaOH contents failed to meet the requirement. Therefore, mixtures with a high water-to-solid ratio and a low relative NaOH content were excluded from subsequent mix-proportion selection.

#### 3.2.3. Stone Formation Rate Analysis

**Figure 5:**
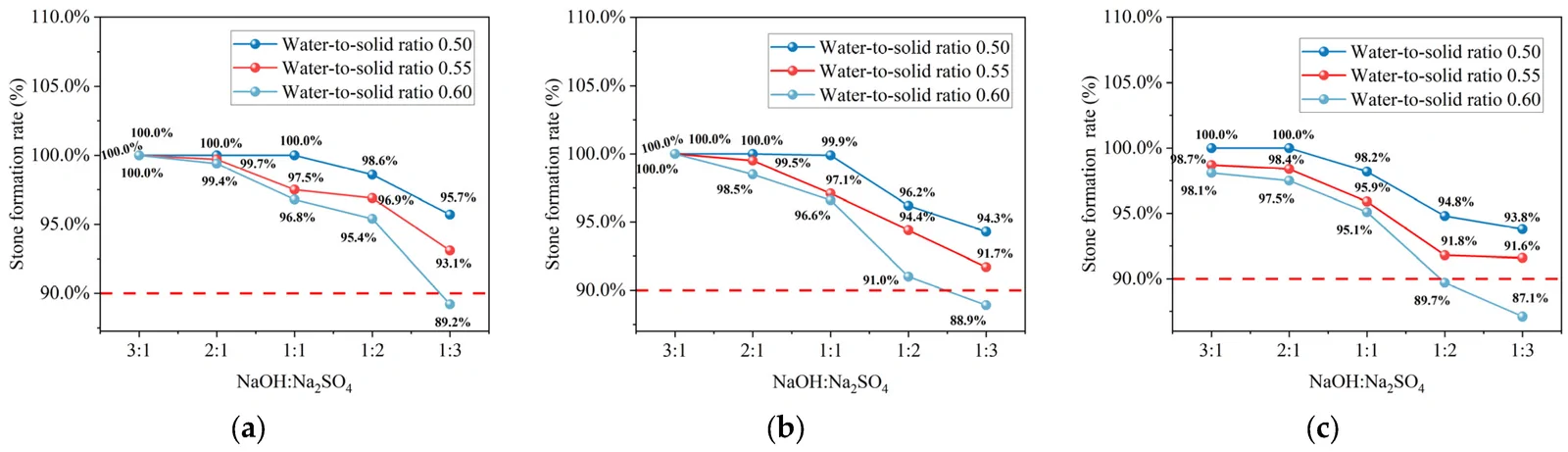
Stone formation rate of the fly ash-based grouting materials: (**a**) total activator dosage of 6%; (**b**) total activator dosage of 5%; (**c**) total activator dosage of 4%.

The stone formation rate variation of the fly ash-based grouting materials under different mix-proportion conditions is shown in Figure 5. The variation pattern of the stone formation rate was generally opposite to that of the bleeding rate. At fixed total activator dosage and NaOH:Na_2_SO_4_ ratio, the stone formation rate decreased with increasing water-to-solid ratio, especially at a water-to-solid ratio of 0.60. This indicates that an excessively high water-to-solid ratio improved fluidity but reduced volume retention after hardening. This reduction was associated with the increased amount of free water and the reduced relative concentration of solid components, which weakened the formation of a compact hardened structure.

The total activator dosage and NaOH:Na_2_SO_4_ ratio also affected the stone formation rate. A lower total activator dosage or lower relative NaOH content was generally associated with a lower stone formation rate, consistent with the bleeding-rate trends. According to the screening requirement that the stone formation rate should not be lower than 90%, mixtures with a water-to-solid ratio of 0.60 and a NaOH:Na_2_SO_4_ ratio of 1:3 were excluded from subsequent mix-proportion selection. When the total activator dosage was 4% and the NaOH:Na_2_SO_4_ ratio was 1:2, the stone formation rate also failed to meet the requirement. Overall, mixture selection should consider fluidity, bleeding rate, and stone formation rate together rather than relying on a single indicator.

### 3.3. Compressive Strength and Mix-Proportion Selection

**Figure 6:**
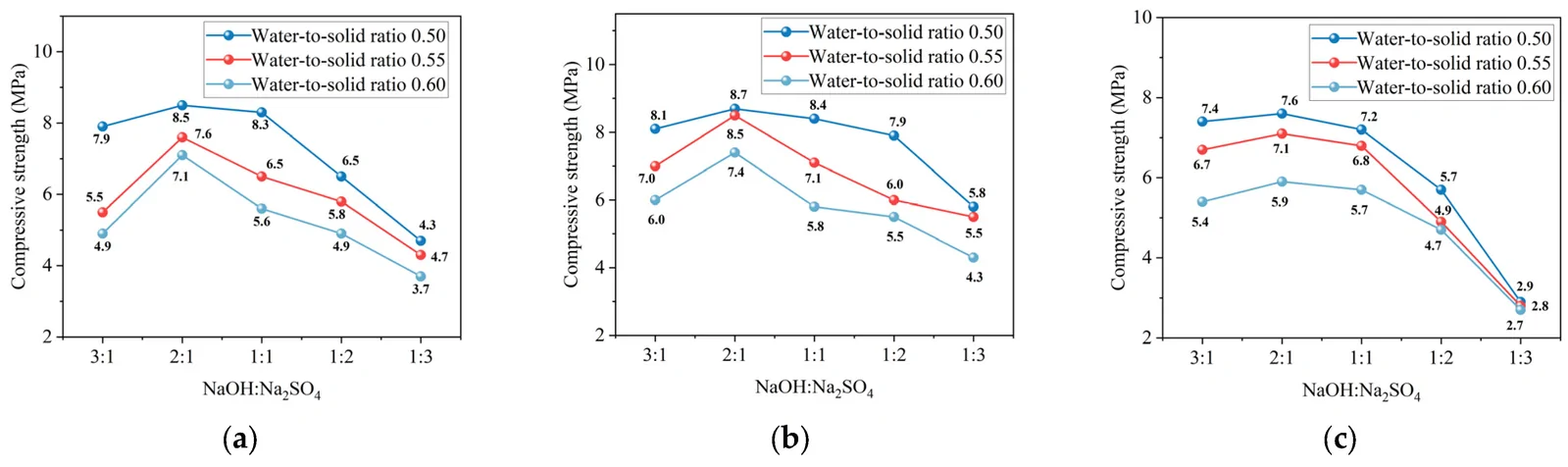
Variation in 7 d compressive strength of fly ash-based grouting materials: (**a**) total activator dosage of 6%; (**b**) total activator dosage of 5%; (**c**) total activator dosage of 4%.

**Figure 7:**
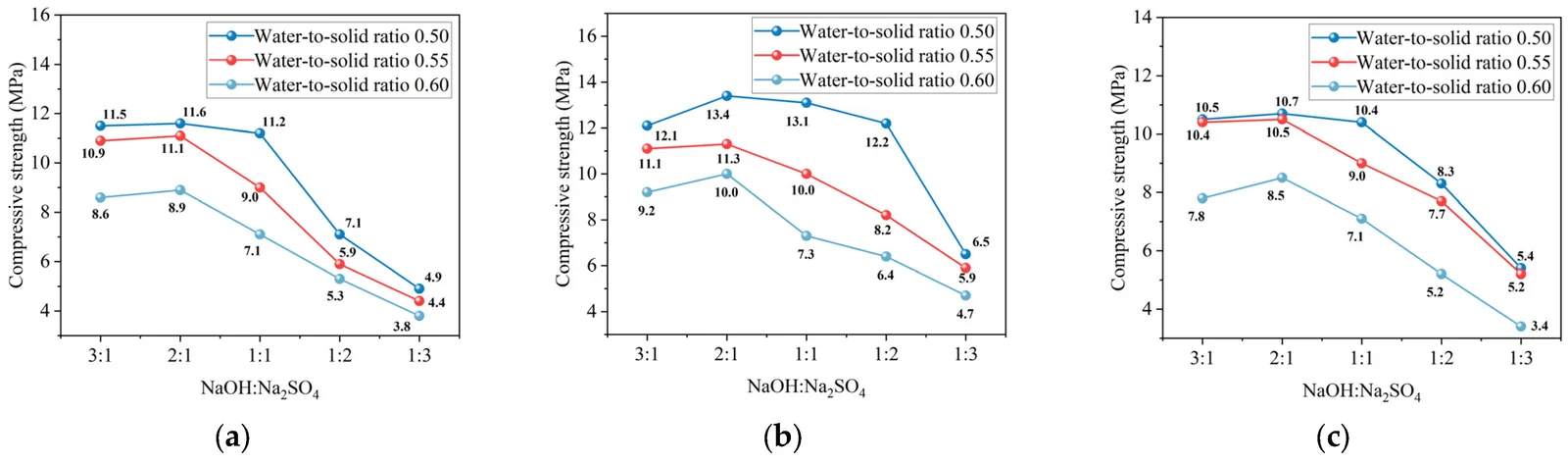
Variation in 28 d compressive strength of fly ash-based grouting materials: (**a**) total activator dosage of 6%; (**b**) total activator dosage of 5%; (**c**) total activator dosage of 4%.

The variations in the 7 d and 28 d compressive strengths of the fly ash-based grouting materials are shown in Figure 6 and Figure 7. The 28 d compressive strength of each mixture was higher than its 7 d strength, indicating continued reaction of the fly ash-GGBFS system under NaOH-Na_2_SO_4_ activation. At fixed total activator dosage and NaOH:Na_2_SO_4_ ratio, the compressive strength increased as the water-to-solid ratio decreased. For example, at a total activator dosage of 5% and a NaOH:Na_2_SO_4_ ratio of 2:1, decreasing the water-to-solid ratio from 0.60 to 0.55 and 0.50 increased the 28 d compressive strength from 10.0 MPa to 11.3 MPa and 13.4 MPa, respectively. A lower water-to-solid ratio increased the solid and activator concentrations per unit volume and reduced the pores formed by the loss of excess water, resulting in closer contact between particles and reaction products. However, a low water-to-solid ratio also reduced fluidity, indicating a trade-off between grout fluidity and compressive strength. Therefore, an appropriate water-to-solid ratio is required to achieve sufficient grout transportability while maintaining adequate hardened strength for practical applications. Similar trends have been reported for alkali-activated grouting materials [9,10].

The effect of total activator dosage on compressive strength was not monotonic. At a water-to-solid ratio of 0.55 and a NaOH:Na_2_SO_4_ ratio of 2:1, the 28 d compressive strengths at total activator dosages of 4%, 5%, and 6% were 10.5 MPa, 11.3 MPa, and 11.1 MPa, respectively. A similar trend was observed at a water-to-solid ratio of 0.50, where the corresponding strengths were 10.7 MPa, 13.4 MPa, and 11.6 MPa. Within the investigated range, a total activator dosage of 5% was generally more favorable for strength development. This non-monotonic response is consistent with reports that activator composition and alkalinity affect reaction progress, pore structure, and compressive strength in alkali-activated GGBFS and multi-solid-waste grouts [27]. An appropriate activator dosage may promote precursor dissolution and reaction-product formation, whereas excessive activator dosage does not necessarily result in continuous strength improvement because the development of a dense structure depends on the balance between reaction progress and pore formation.

The NaOH:Na_2_SO_4_ ratio also affected compressive strength. At a water-to-solid ratio of 0.55 and a total activator dosage of 5%, the 28 d compressive strengths corresponding to NaOH:Na_2_SO_4_ ratios of 3:1, 2:1, 1:1, 1:2, and 1:3 were 11.1 MPa, 11.3 MPa, 10.0 MPa, 8.2 MPa, and 5.9 MPa, respectively. At a water-to-solid ratio of 0.50, the corresponding strengths were 12.1 MPa, 13.4 MPa, 13.1 MPa, 12.2 MPa, and 6.5 MPa. In most cases, NaOH:Na_2_SO_4_ = 2:1 produced the highest compressive strength, indicating that an appropriate NaOH:Na_2_SO_4_ ratio was beneficial for balancing alkaline dissolution of aluminosilicate components and sulfate-related reaction-product formation.

Mix-proportion screening was conducted by comprehensively considering the results of fluidity, bleeding rate, stone formation rate, and compressive strength. Based on this screening, specimen A17, with a water-to-solid ratio of 0.55, a total activator dosage of 5%, and NaOH:Na_2_SO_4_ = 2:1, was selected. Its fluidity was 195.0 mm, bleeding rate was 1.70%, stone formation rate was 99.50%, and 7 d and 28 d compressive strengths were 8.5 MPa and 11.3 MPa, respectively. This mixture met all three screening criteria and provided balanced overall performance in terms of fluidity, bleeding rate, stone formation rate, and compressive strength. This mixture achieved a balance between fresh-state performance and hardened strength, providing a suitable basis for the practical application of fly ash-based grouting materials.

### 3.4. Microstructure and Phase Analysis

To examine the microstructural differences associated with NaOH:Na_2_SO_4_, representative 7 d specimens were selected for SEM, EDS, and XRD analyses. The water-to-solid ratio and total activator dosage were fixed at 0.55 and 5%, respectively, and specimens with NaOH:Na_2_SO_4_ ratios of 3:1, 2:1, and 1:1 were compared. These ratios included the preferred ratio and adjacent comparison levels with higher and lower relative NaOH contents.

**Figure 8:**
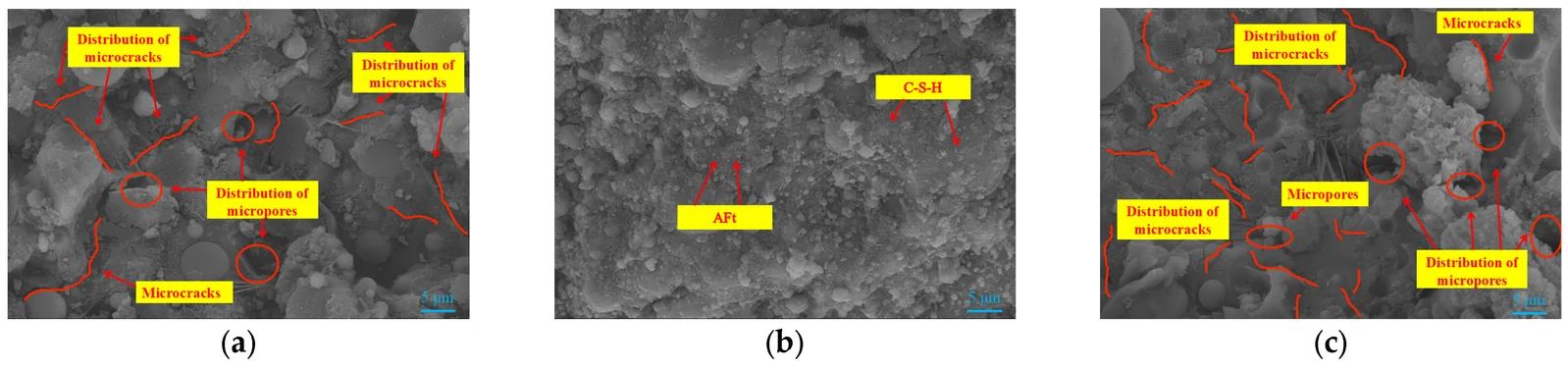
SEM images of specimens with different NaOH:Na_2_SO_4_ ratios: (**a**) NaOH:Na_2_SO_4_ = 3:1; (**b**) NaOH:Na_2_SO_4_ = 2:1; (**c**) NaOH:Na_2_SO_4_ = 1:1.

As shown in Figure 8, the specimens exhibited clear differences in pore, crack, and reaction-product morphology. At NaOH:Na_2_SO_4_ = 3:1, local micropores and microcracks remained, and particle bonding was incomplete. At NaOH:Na_2_SO_4_ = 2:1, the specimen exhibited a more uniform and compact structure with fewer pores and cracks. Gel-like products and needle- or column-like crystalline features were observed. These morphologies were consistent with C-S-H-type phases and AFt-related products reported in previous studies. At NaOH:Na_2_SO_4_ = 1:1, the number of micropores and microcracks increased, and the structure became less compact. These observations suggest that NaOH:Na_2_SO_4_ affected the extent and spatial distribution of reaction products. A similar influence of NaOH addition on the reaction and microstructure of Na_2_SO_4_-activated GGBFS has been reported [27].

The local EDS results are presented in Table 3. The analyzed regions exhibited Ca/Si ratios of 0.73, 0.94, and 1.86 at NaOH:Na_2_SO_4_ ratios of 3:1, 2:1, and 1:1, respectively. At NaOH:Na_2_SO_4_ = 2:1, the analyzed region had a Ca/Si ratio of 0.94 and the highest O content. Together with the denser SEM morphology, this local composition suggests the possible presence of Ca-containing reaction products in the analyzed region. The higher Ca/Si ratio at NaOH:Na_2_SO_4_ = 1:1 was accompanied by more pores and cracks, indicating that local Ca enrichment in the analyzed region was not necessarily associated with a denser microstructure. Because EDS was performed on selected regions, these values should be interpreted as local compositional indicators rather than bulk elemental compositions.

**Figure 9:**
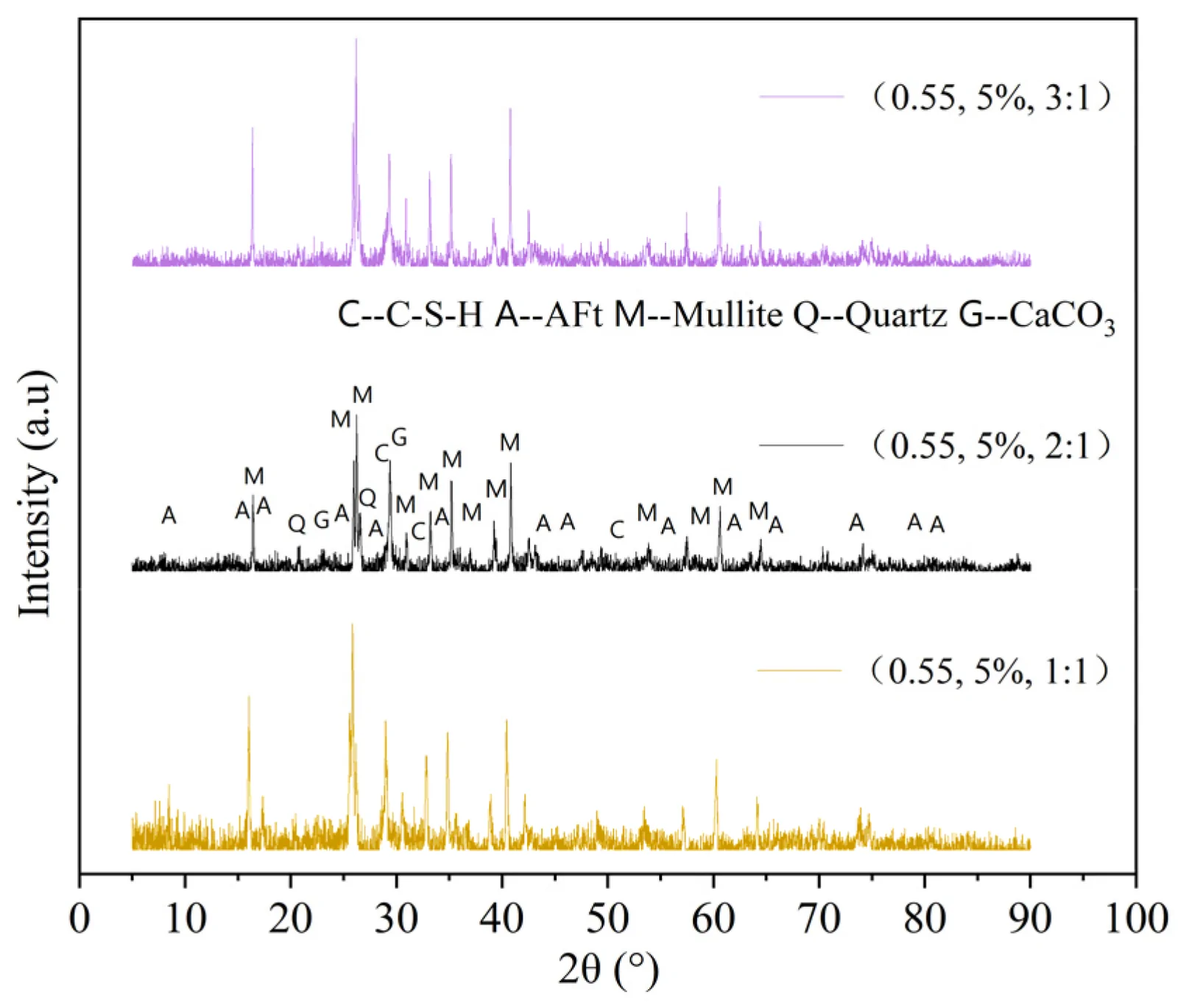
XRD patterns of specimens with different NaOH:Na_2_SO_4_ ratios.

As shown in Figure 9, mullite and quartz were detected in specimens with different NaOH:Na_2_SO_4_ ratios, indicating that part of the stable crystalline phases in the fly ash remained unreacted at 7 d. Diffraction features associated with AFt and CaCO_3_ were observed, while the gel-related diffraction features marked in Figure 9 were interpreted in conjunction with the SEM and EDS results because of their relatively low crystallinity. Compared with the specimens at NaOH:Na_2_SO_4_ ratios of 3:1 and 1:1, the specimen at NaOH:Na_2_SO_4_ = 2:1 exhibited relatively more pronounced AFt-related features and changes in the diffraction features of the residual crystalline phases, indicating differences in reaction extent and phase assemblage. These phase characteristics were consistent with the denser morphology and higher compressive strength observed at NaOH:Na_2_SO_4_ = 2:1. Gel products and AFt may contribute to particle bonding and pore filling in the hardened grout.

Overall, the SEM, EDS, and XRD results showed that different NaOH:Na_2_SO_4_ ratios resulted in distinct microstructural characteristics in the fly ash-based grouting materials, which were closely related to their compressive strength. At NaOH:Na_2_SO_4_ = 2:1, the formation and distribution of reaction products contributed to a denser structure with fewer pores and cracks, which was consistent with the higher compressive strength observed at this ratio. When NaOH:Na_2_SO_4_ deviated from 2:1, less favorable reaction-product formation and pore filling resulted in a less compact structure, corresponding to lower compressive strength.

## 4. Conclusions

In this study, fly ash and GGBFS were used as the main binder components, and fly ash-based grouting materials were prepared through NaOH-Na_2_SO_4_ composite activation. The effects of water-to-solid ratio, total activator dosage, and NaOH:Na_2_SO_4_ on grout workability and compressive strength were investigated, and the microstructural characteristics at representative NaOH:Na_2_SO_4_ ratios were analyzed. The following conclusions can be drawn:

1. Increasing the water-to-solid ratio increased grout fluidity but also increased the bleeding rate and reduced the stone formation rate and compressive strength. Reducing the total activator dosage or the relative NaOH content generally increased both fluidity and the bleeding rate but reduced the stone formation rate.
2. Within the investigated experimental range, an appropriate activator dosage and NaOH:Na_2_SO_4_ ratio were important for achieving high compressive strength. The combination of 5% total activator dosage and NaOH:Na_2_SO_4_ = 2:1 provided favorable conditions for strength development.
3. Considering fluidity, bleeding rate, stone formation rate, and compressive strength together, the mixture with a water-to-solid ratio of 0.55, a total activator dosage of 5%, and NaOH:Na_2_SO_4_ = 2:1 was selected as the preferred mixture within the investigated experimental range.
4. The microstructural analysis indicated that NaOH:Na_2_SO_4_ = 2:1 promoted a denser internal structure with fewer visible pores and cracks, which was consistent with the higher compressive strength obtained under this condition.

The results demonstrate the feasibility of using NaOH-Na_2_SO_4_ composite activation to prepare fly ash-based grouting materials with balanced workability and mechanical performance, providing an experimental basis for mix-proportion design and potential engineering application.

Further research should extend the present study to setting behavior, strength prediction, and the mechanical response of grout-reinforced gangue, together with long-term durability and engineering-scale performance under mining conditions. Long-term performance evaluation, including extended-age strength development, freeze–thaw resistance, and volume stability, is also needed to further assess the potential engineering application of the proposed grouting materials.

## Figures and Tables

**Table 1:** Mix proportions and mixture IDs of the fly ash-based grouting materials.

Mixture ID	Mix Proportion			Mixture ID	Mix Proportion			Mixture ID	Mix Proportion		
	Water-to-Solid Ratio	Activator			Water-to-Solid Ratio	Activator			Water-to-Solid Ratio	Activator	
		Dosage	NaOH:Na_2_SO_4_			Dosage	NaOH:Na_2_SO_4_			Dosage	NaOH:Na_2_SO_4_
A1	0.55	6%	3:1	A16	0.55	5%	3:1	A31	0.55	4%	3:1
A2	0.55	6%	2:1	A17	0.55	5%	2:1	A32	0.55	4%	2:1
A3	0.55	6%	1:1	A18	0.55	5%	1:1	A33	0.55	4%	1:1
A4	0.55	6%	1:2	A19	0.55	5%	1:2	A34	0.55	4%	1:2
A5	0.55	6%	1:3	A20	0.55	5%	1:3	A35	0.55	4%	1:3
A6	0.60	6%	3:1	A21	0.60	5%	3:1	A36	0.60	4%	3:1
A7	0.60	6%	2:1	A22	0.60	5%	2:1	A37	0.60	4%	2:1
A8	0.60	6%	1:1	A23	0.60	5%	1:1	A38	0.60	4%	1:1
A9	0.60	6%	1:2	A24	0.60	5%	1:2	A39	0.60	4%	1:2
A10	0.60	6%	1:3	A25	0.60	5%	1:3	A40	0.60	4%	1:3
A11	0.50	6%	3:1	A26	0.50	5%	3:1	A41	0.50	4%	3:1
A12	0.50	6%	2:1	A27	0.50	5%	2:1	A42	0.50	4%	2:1
A13	0.50	6%	1:1	A28	0.50	5%	1:1	A43	0.50	4%	1:1
A14	0.50	6%	1:2	A29	0.50	5%	1:2	A44	0.50	4%	1:2
A15	0.50	6%	1:3	A30	0.50	5%	1:3	A45	0.50	4%	1:3

**Table 2:** Chemical composition of the fly ash.

Chemical component	SiO_2_	Al_2_O_3_	Fe_2_O_3_	CaO	TiO_2_	SO_3_	K_2_O	Others
Content (wt%)	50.397	38.823	3.713	1.832	1.278	1.208	1.124	1.625

**Table 3:** Local EDS results for specimens with different NaOH:Na_2_SO_4_ ratios.

NaOH:Na_2_SO_4_	Elemental Mass Fraction (wt%)				Ca/Si
	Si	Al	O	Ca	
3:1	16.85	12.08	41.84	12.32	0.73
2:1	14.09	10.24	45.85	13.18	0.94
1:1	13.80	7.99	26.33	25.61	1.86

## Data Availability

The original contributions presented in this study are included in the article. Further inquiries can be directed to the corresponding author.
